# Neurostimulation on lumbosacral nerves as a new treatment for spinal cord injury impairments and its impact on cortical activity: a narrative review

**DOI:** 10.3389/fnhum.2024.1478423

**Published:** 2024-12-13

**Authors:** Rodrigo Lantyer Marques Dantas, Diego N. Vilela, Mariana Cardoso Melo, Gustavo Fernandes, Nucelio Lemos, Jean Faber

**Affiliations:** ^1^Neuroscience Division, Department of Neurology and Neurosurgery, Escola Paulista de Medicina, Federal University of São Paulo, São Paulo, Brazil; ^2^Biomedical Engineering Division, Institute of Science and Technology, Federal University of São Paulo, São José dos Campos, Brazil; ^3^Department of Gynecology, Federal University of São Paulo, São Paulo, Brazil; ^4^Department of Gynecology and Neuropelveology, Increasing-Institute of Care and Rehabilitation in Neuropelveology and Gynecology, São Paulo, Brazil; ^5^Department of Obstetrics and Gynecology, Santa Casa de São Paulo School of Medical Sciences, São Paulo, Brazil; ^6^Department of Obstetrics and Gynecology, Faculty of Medicine, University of Toronto, Toronto, ON, Canada

**Keywords:** neuromodulation, spinal cord injury, laparoscopic neuroprosthesis, sensorimotor rehabilitation, neural stimulation

## Abstract

Spinal cord injury (SCI) can cause significant motor, sensory, and autonomic dysfunction by disrupting neural connections. As a result, it is a global health challenge that requires innovative interventions to improve outcomes. This review assesses the wide-ranging impacts of SCI and focuses on the laparoscopic implantation of neuroprosthesis (LION) as an emerging and promising rehabilitation technique. The LION technique involves the surgical implantation of electrodes on lumbosacral nerves to stimulate paralyzed muscles. Recent findings have demonstrated significant improvements in mobility, sexual function, and bladder/bowel control in chronic SCI patients following LION therapy. This manuscript revisits the potential physiological mechanisms underlying these results, including neuroplasticity and modulation of autonomic activity. Additionally, we discuss potential future applications and amendments of LION therapy. This study emphasizes the potential of neuromodulation as a complementary approach to traditional rehabilitation, that can provide a beacon of hope for improving functionality and quality of life for individuals with SCI.

## Introduction

Spinal cord injuries (SCI) are debilitating conditions that significantly impair quality of life and demand specialized care and innovative treatment approaches (Ahuja et al., 2017; Alizadeh et al., 2019; Ambrozaitis et al., 2006). According to 2016 data, the incidence of SCI is substantial, affecting 13 (11 to 16) individuals per 100,000 residents globally (Safdarian et al., 2023; GBD 2019 Diseases and Injuries Collaborators, 2020). These indices show how SCI impacts a notable amount of people globally.

The etiology of SCI can be categorized into traumatic, caused by external forces, and non-traumatic, resulting from neurodegenerative and/or ischemic processes, each leading to potentially irreversible spinal cord damage (Fehlings et al., 2017; Rouanet et al., 2017). Understanding the etiology of SCI is crucial for developing effective treatments and interventions for individuals with spinal cord injuries.

In a SCI, a physical interruption of neural connections directly affects the flow of information in the cortico-motor circuit, leading to motor, sensory, and autonomic dysfunctions, which will be more severe the more proximal and more extensive the injury is (Ahuja et al., 2017; Alizadeh et al., 2019). The most common impairments are spasticity, rigidity, hypotonicity, spasms, loss of control, phantom sensations, itches, and loss of sensibility (Rouanet et al., 2017; Perrouin-Verbe et al., 2021; Guest et al., 2022). The American Spinal Cord Injury Association (ASIA) quantifies the severity of the injury through the ASIA Impairment Scale (AIS). Complete injuries (AIS A) are characterized by complete interruption of motoric and sensory communication at the level of the injury. In motoric complete injuries (AIS B), sensory signals are still transmitted, but no voluntary motor function can be found below the level of injury. While, in incomplete injuries (AIS C-D), motoric and sensory functions are partially preserved below the level of injury (Fehlings et al., 2017; Nandoe Tewarie et al., 2010).

In the early stages of spinal cord injury, known as spinal shock phase, there is a reduction or complete abolition of the conduction of impulses (Rogers and Todd, 2016). During the chronic phase, the formation of the glial scars acts as a physical obstruction to axonal growth, leading to a reduction in functionality proportional to the extent of the injury (Atkinson and Atkinson, 1996; Ashby et al., 1974; Ditunno et al., 2004). Additionally, the autonomic effects can vary greatly from the acute to chronic phases, ranging from autonomic dysreflexia to hemodynamic instability, which may have negative implications for cardiovascular function and neurological recovery, thereby endangering both survival and quality of life (Eldahan and Rabchevsky, 2018; Spinal Cord Injury Centre of Western Denmark, 2021; Wulf and Tom, 2023; Goudman et al., 2022).

After a SCI, the thalamus recalibrates the integration of sensory and motor inputs, potentially altering the synchronization dynamics of the entire circuit (Alonso-Calviño et al., 2016; Murray Sherman and Guillery, 2002). This can lead to disruptions in the functioning of the cortico-thalamic system, resulting in impairments in behavior or neurocognitive functions in the long term. Afferent fibers play a crucial role in providing sensory feedback for precise movement control. Any delay in this signal, caused by partial or total deafferentation in the spinal cord, can lead to desynchronization of electrophysiological activity in the central nervous system regions responsible for decoding movement. It is important to note that this disruption can have significant consequences on movement coordination and control (Alonso-Calviño et al., 2016; Murray Sherman and Guillery, 2002; Murray Sherman, 2001).

Besides motor impairments, recent clinical studies have confirmed that patients with SCI also exhibit symptoms of attention decrease, loss of concentration and memory, and learning deficits (Distel et al., 2020; Molina et al., 2018; Sachdeva et al., 2018), with compromising effects on medial prefrontal cortex, and anterior cingulate, which are critical regions for emotional processing and attention modulation (Molina et al., 2018; Bouton et al., 2016; Moxon et al., 2014). Additionally, epidemiological studies suggest that SCI patients are at a heightened risk for developing dementia and mood disorders such as depression, anxiety, and PTSD (Perrouin-Verbe et al., 2021; Li et al., 2020; Budd et al., 2022; Huang et al., 2017).

Up to this moment, SCIs are only partially reversible, depending on the type and extension of the lesion (Rouanet et al., 2017; Rath and Balain, 2017; Flack et al., 2022). However, many new neurostimulation procedures are currently showing great progress and promising results in the medium term. These neurostimulation protocols can be used in conjunction with traditional rehabilitation therapies, such as physical therapy and occupational therapy (Carè et al., 2024; Capogrosso et al., 2018; Chalif et al., 2024; Alam et al., 2016). They can help to promote neuroplasticity (Bouton et al., 2016) or assist respiratory pacing and bladder control (Stampas et al., 2019; de Cássia Meine Azambuja et al., 2018), still allowing volitional and functional movements (Bouton et al., 2016; Grahn et al., 2014; James et al., 2018).

A promising neuromodulation technique is the LION (Laparoscopic Implantation of Neuroprosthesis) procedure^1^—the implantation of four stimulation electrodes onto the sciatic, pudendal, and femoral nerves bilaterally, allowing for continuous and on demand stimulation of those nerves (Possover, 2022; Possover, 2009; Possover et al., 2007a).

Recently, Lemos et al. (2023) assessed the impact of the LION procedure on mobility, sexual, urinary, and anorectal functions of 30 subjects with chronic spinal cord injury (SCI), showing that the procedure improves mobility and genital sensitivity and reduces the number of urinary and fecal incontinence episodes (Spinal Cord Injury Centre of Western Denmark, 2021). These results reinforced the establishment of neuromodulation with the LION procedure as an additional therapeutic resource for rehabilitating patients with chronic SCI.

On this narrative review, we will broadly evaluate the possible physiological mechanisms involved during neuromodulation-augmented rehabilitation strategies. We will then analyze the main LION neuromodulation findings and discuss the implications, perspectives, and limitations regarding the results, in light of the overall understanding built on neuromodulation in general. Finally, we will assess possible extensions of this technique and propose directions for future research.

## Neuromodulation protocols for SCI patients

In this manuscript, we will use the term neuromodulation as any deliberate modulation of the nervous system’s activity through the application of electrical current, voltage difference, or directed magnetic/electric field near or onto neuronal cells (Carè et al., 2024; Deer et al., 2017; Sun and Morrell, 2014).

Neuromodulation can be either invasive or non-invasive. Invasive neuromodulation involves the surgical implantation of electrodes into the brain, spinal cord, nerve roots or peripheral nerves; while non-invasive involves external devices that stimulate the nervous system from outside the body (Capogrosso et al., 2018; Sun and Morrell, 2014; Kumar et al., 2005). Semi-invasive neuromodulation refers to methods involving simpler electrode implantation procedures, typically using an external generator (Dolbow et al., 2021). Table 1 provides an overview of some types of neuromodulations (protocols and devices). The main techniques are deep brain stimulation (DBS) (Gielen and Molnar, 2012; Benabid et al., 2009; Vachez and Creed, 2020), Vagus nerve stimulation (VNS) (Goudman et al., 2022; Terra et al., 2013; Rush et al., 2005), transcranial magnetic stimulation (TMS) (Alexeeva and Calancie, 2016; Rossi et al., 2021; Rossi et al., 2009), transcranial direct current stimulation (tDCS) (Thair et al., 2017; Arora et al., 2022; Li et al., 2021), spinal cord stimulation (SCS) (Chalif et al., 2024; Grahn et al., 2014; Kumar et al., 2005; Li et al., 2021; Gad et al., 2013), surface functional electrical stimulation (sFES) (Peckham and Knutson, 2005; Popović, 2014; Selfslagh et al., 2019; Westerveld et al., 2013), peripheral nerve stimulation (PNS) (Abd-Elsayad and Trescot, 2022; St Clair et al., 2003; Rossignol et al., 2008), neuromuscular electrical stimulation (NMES) (de Cássia Meine Azambuja et al., 2018; Lake, 1992; Ethier et al., 2015) and, more recently, LION (Spinal Cord Injury Centre of Western Denmark, 2021; Possover, 2022; Lemos et al., 2023; Lemos and Possover, 2015). These neuromodulation techniques are employed in diverse applications, treating conditions ranging from chronic pain and Parkinson’s disease to muscle spasticity and various psychiatric disorders (Benabid et al., 2009; Terra et al., 2013; Deer et al., 2019; Hofstoetter et al., 2014; Taylor et al., 2018).

**Table 1:** Description of neuromodulation techniques alongside their pros and cons.

Neuromodulation device	Description	Pros	Cons
Surface functional electrical stimulation (sFES)	Electrical stimulation applied to the skin surface to activate muscles and improve motor function	Non-invasive Improves muscle strength and coordination in SCI patients with incomplete injuries (Peckham and Knutson, 2005) Enhances functional activities such as hand grasp (Doucet et al., 2012)	Limited control over specific muscle groups (e.g., precise targeting difficult due to surface application) Ineffective in complete SCI cases (Peckham and Knutson, 2005)
Transcranial magnetic stimulation (TMS)	Magnetic fields applied to the scalp to induce electric currents in the brain, modulating neural activity	Non-invasive Effective in reducing spasticity and enhancing motor cortex plasticity in post-stroke rehabilitation (Lefaucheur et al., 2020) Targeting of motor cortex can improve motor function in some SCI cases (Nielsen, 2002)	Limited depth of penetration, typically reaches only superficial cortical layers Skilled operators required to achieve consistent results Effects often temporary and variable across studies (Lefaucheur et al., 2020)
Peripheral nerve stimulation (PNS)	Electrical stimulation applied directly to peripheral nerves to modulate neural activity	Target-specific, effective for peripheral nerves associated with pain relief (Deer et al., 2016) Long-term benefits observed for neuropathic pain in some cases (Ilfeld et al., 2019)	Invasive, requiring surgical implantation Risk of infection or nerve damage in ~10% of cases (Deer et al., 2016) Limited long-term data on effectiveness for motor function improvement
Muscular electrical stimulation (MES)	Electrical stimulation applied directly to muscles (semi invasively or non-invasively) to improve strength and function	Improves muscle strength and endurance in patients with muscular atrophy (Bax et al., 2005) Effective for specific muscle targeting to aid motor rehabilitation (Snyder-Mackler et al., 1994)	Limited control over fine movements, especially for hand muscles (Bax et al., 2005) Fatigue reported in up to 30% of sessions (Snyder-Mackler et al., 1994) Not suitable for complete SCI due to lack of neural connection
Transcranial direct current stimulation (tDCS)	Low electrical currents applied to the scalp to modulate cortical excitability	Non-invasive and relatively easy to apply Shows potential in enhancing motor learning tasks in stroke and SCI patients (Dedoncker et al., 2016)	Effects highly variable; optimal parameters (intensity, duration) are not fully established (Dedoncker et al., 2016) Effects are often transient, with benefits lasting hours to days post-treatment (Nitsche and Paulus, 2000)
Invasive spinal cord stimulation (iSCS)	Electrical stimulation applied to the spinal cord to modulate neural activity and alleviate pain	Well-documented pain relief for chronic neuropathic pain (North et al., 1996) Shows functional improvement in some SCI cases, specifically in lower limb strength (Kriek et al., 2022)	Invasive procedure requiring surgical implantation Side effects like discomfort or paresthesia in 15% of patients (Kriek et al., 2022) Limited evidence for motor function improvement in cases of complete SCI
Non-invasive spinal cord stimulation (nSCS)	Transcutaneous electrical stimulation applied over skin directed to the spinal cord to modulate neural activity	Effective for pain relief in chronic pain patients without invasive surgery (Estores et al., 2021) Observed improvements in motor function in incomplete SCI patients, with ~20% showing progress (Yadav and Ahmed, 2018)	Potential for discomfort and paresthesia in ~10% of sessions (Yadav and Ahmed, 2018) Limited specificity in targeting due to skin application
Laparoscopic implantation of neuroprosthesis (LION)	Laparoscopic implantation of neuroprosthesis to modulate neural activity	Targeted neural modulation, showing promise in SCI cases with retained sensory pathways Minimally invasive with quicker recovery time than open surgery (Possover and Forman, 2015)	Limited long-term data; lack of extensive clinical trials for motor improvement (Possover and Forman, 2017) Complex procedure, requiring specialized training and carries risks associated with laparoscopy (Lemos et al., 2023; Possover and Forman, 2015)

Considering sensorimotor conditions, currently, two important new protocols use epidural spinal stimulation (Chalif et al., 2024; Choi et al., 2021; Greiner et al., 2021) (with surgical implantation of electrodes above the dorsal surface of the spinal cord) or transcutaneous spinal cord stimulation (Hofstoetter et al., 2014; Hofstoetter et al., 2020; Shkorbatova et al., 2020) (with non-invasive electrodes placement the patient’s skin over the vertebral column) to enhance spinal excitability. These methods have shown promising results in restoring voluntary motor output in SCI and post-stroke patients (Owolabi et al., 2022; Taylor et al., 2021).

Direct brain stimulation approaches modulate supraspinal circuits by increasing descending activity. They have also shown improvements onto upper and lower limbs motor output after SCI (Rossi et al., 2009; Arora et al., 2022; Li et al., 2021; Nitsche and Paulus, 2000). TMS and tDCS are the most used techniques for this purpose. TMS is a non-invasive brain stimulation technique that induces directed magnetic fields through the scalp and skull, evoking neuro-electrophysiological responses. Although most studies using repetitive TMS in individuals with para/tetraplegia have a small sample sizes, the results are promising (Alexeeva and Calancie, 2016; Rossi et al., 2021). Stimulation of the peripheral nervous system can be performed by semi-invasive procedures, using PNS and NMES (Carè et al., 2024; Deer et al., 2017; Abd-Elsayad and Trescot, 2022; St Clair et al., 2003), or non-invasive using sFES (Peckham and Knutson, 2005; Popović, 2014; Teferra, 2017). These techniques are currently the most established, clinically accessible, and reliable form of neuromodulation for SCI patients (with many devices commercially available and used in the clinic) (James et al., 2018; Wenger et al., 2014). Finally, the LION protocol brought a new perspective by stimulating the femoral, sciatic, and pudendal nerves of SCI patients. By associating a systematic activation of residual pathways through pre-parametrized electric stimuli on pelvic nerves, conjugated with standard rehabilitation procedures, the technique showed to be very promising (Spinal Cord Injury Centre of Western Denmark, 2021; Lemos et al., 2023).

## Stimulation effects on lumbosacral roots

The lumbosacral plexus comprises the nerves of the lumbar (L1–L5) and sacral (S1–S5) plexuses, which supports all motor and sensory innervation of the lower extremity, as well as the pelvic floor and abdominal muscles, the kidneys, bladder, sexual organs, colon, and rectum through both somatic and autonomic nerve pathways (Moore et al., 2013; Harkema et al., 2011; Possover et al., 2007c).

Possover et al. (2007a,b) described the first laparoscopic implantation of electrodes onto the intrapelvic portion of lumbosacral nerves for treating refractory pelvic neuropathic pain and dysfunction of the urinary tract and bladder, naming it as the LION procedure. Since then, this procedure has been adapted to improve bowel function, sexual function, and gait in patients with SCI (Possover, 2022; Lemos and Possover, 2015).

The LION protocols comprise a personalized set of programs to be used in specific situations of patient daily life. The fundamental programming encompasses a low-frequency baseline program of stimulus (5–20 Hz), continually operative throughout the day to inhibit detrusor overactivity, improve baseline muscle tone and modulate spasticity of the muscles of the lower limbs, while stimulating neuroplasticity in the mid-long term (Harkema et al., 2011; Brindley, 1974; Billington et al., 2022). Individual mid-frequency protocols (20–40 Hz) are activated at the patient’s request to promote intermittent contractions of the quadriceps, deep gluteal, and pelvic floor muscles for the purposes of training and physiotherapy (Lemos and Possover, 2015; Laufer et al., 2001). In addition, there is a continuous stimulation protocol for orthostatism and gait training. The patient can use a remote control to select and activate programs as needed. Other programs can occasionally be established upon patient indication or request to aid specific purposes, such as penile erection, enhance the absorption of lower limb oedema, and aid in specific transfer requirements (Spinal Cord Injury Centre of Western Denmark, 2021; Lemos et al., 2023). The lowest frequency to induce a harmonic contraction (without tremor/vibration) was used for the programs stimulating the femoral, sciatic and gluteus maximus nerves, as higher frequencies induce rapid onset of muscle fatigue (Possover et al., 2007c; Laufer et al., 2001; Lemos et al., 2018; Lemos et al., 2021).

**Figure 1:**
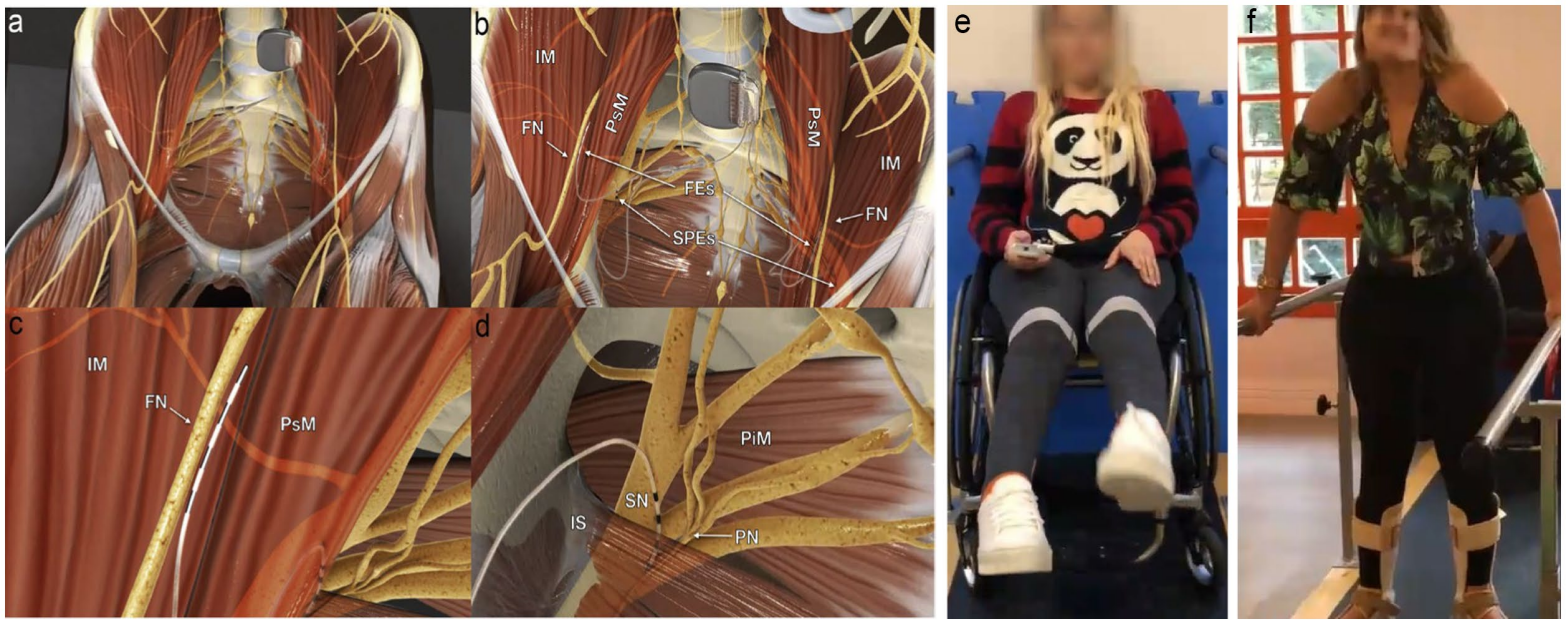
Electrodes placement and nerve stimulation. **(A)** Panoramic view of the system; the pulse generator is implanted into a paraumbilical subcutaneous pocket, and the electrodes run retroperitoneally down to the intrapelvic portions of the femoral (FN), sciatic (SN), and pudendal nerves (PN) bilaterally. **(B)** Panoramic view of femoral electrodes (FEs) and sciatic and pudendal electrodes (SPEs) positioning. **(C)** Detailed view of the right femoral electrode over the nerve between the iliac (IM) and the psoas (PsM) muscles. **(D)** Detailed view of the right sacral electrodes placed with half of its poles in the Alcock’s canal over the PN and the remaining poles over the SN. IS, ischial spine; PiM, piriformis muscle. **(E)** Extension of knees through femoral nerve stimulation using a remote activation. **(F)** Weight transfer and walking on parallel bars. Figure adapted from Lemos et al. (2023), with permission from the authors.

Frequencies of less than 40 to 50 Hz are known to preferentially activate slow twitch type I muscle fibers, which demonstrate higher resistance to muscle fatigue, while higher frequencies lead to the recruitment of fast twitch type IIa and type IIb muscle fibers, which fatigue easily. To train the muscles of the pelvic floor, the same approach was taken, using lower frequencies (30 Hz) to train the slow fibers and higher frequencies (50 Hz) to train the fast fibers. Pudendal neuromodulation in the range of 10 to 15 Hz was also employed to enhance bladder compliance and reduce neurogenic detrusor overactivity (Lemos and Possover, 2015; Kasch et al., 2022; Lemos et al., 2021; Billot et al., 2020) (see Figure 1).

Additionally, pulse width (the duration of the electrical pulse applied to the muscle) was 60 μs for the femoral and sciatic nerves and 210 μs. The combination of frequency and pulse width is crucial in determining the strength and duration of muscle contraction. In 2022, Lemos et al. (2023) longitudinally assessed the impact of the LION procedure on mobility (Possover, 2014), sexual, urinary, and anorectal functions of 30 people with chronic spinal cord injury (SCI) (Lemos et al., 2023). Through a pre-programmed electrical stimulation protocol, combined with a specific rehabilitation program, they showed a significant improvement of different physiological functions. The most important were:

### Impact on mobility

Lemos et al. (2023) showed that patients who underwent the LION procedure were able to regain walker-assisted gait, especially in their homes. The patients also became less dependent on adapted environments, such as being able to get up and reach objects on shelves or enter a non-adapted shower and sit on a standard chair to take a shower. After 1 year of follow-up, 72% of patients with thoracic injuries and 60% of patients with cervical injuries were able to walk with a walker using specific protocol that was activated on demand. Similarly, a randomized controlled trial (RCT) published by Kasch et al. (2022) further reinforced the potential of the LION procedure in enhancing gait ability. This study observed a significant improvement in the WISCI II scale for patients in the LION group, without changes in the control group. Notably, in this study, the LION participants were more homogeneous in terms of SCI level and injury type focusing on individuals with at least 12 months post-injury. The increase in WISCI II scores was clinically significant, aligning with previous case series reporting improvements in mobility over extended follow-up periods.

### Impact on urinary function

47.8% of patients improved their urinary incontinence category, meaning that they experienced fewer episodes of leakage. The remaining 52.2% remained unchanged, and none of the patients’ incontinence worsened. Nighttime urinary incontinence improved in 30.4% of patients (Lemos et al., 2023). These findings align with evidence from studies on sacral root stimulation, which has been shown to activate bladder function effectively. For instance, the sacral anterior root stimulator (SARS) demonstrated significant reductions in urinary infections, from a median of seven to one per year, in a cohort of 20 participants (Brindley, 1994). Furthermore, 18 of these participants became catheter-free, and detrusor hyperreflexia was successfully abolished, enabling discontinuation of anticholinergic medications and reducing associated side effects such as constipation, dry mouth, and drowsiness. Over 85% of patients achieved continence, largely due to improved bladder compliance following posterior rhizotomy (Brindley, 1994; Creasey et al., 2001; Creasey and Dahlberg, 2001).

### Impact on bowel function

In Lemos et al. (2023) study, before the surgery, 52% of patients needed more than 30 min to have a bowel movement. At follow-up, 65% of patients had reduced their bowel routine to less than 30 min. These results are consistent with findings from SARS studies, where bowel management was significantly enhanced (Creasey and Dahlberg, 2001). In these studies, the volunteers reported reduction of constipation and decreased reliance on laxatives and stool softeners. Additionally, bowel emptying time was reduced by 75%, offering substantial patient comfort. The intermittent stimulation used for micturition was adapted successfully for bowel evacuation, demonstrating the versatility of sacral stimulation in addressing multiple physiological functions (Creasey and Dahlberg, 2001).

### Impact on sexual function

The International Index for Erectile Function Questionnaire showed improvement in erection in male patients. Additionally, 71% of patients of both sexes reported improved sensitivity in the genital area (Lemos et al., 2023).

Although these results show great improvements in a range of physiological functions for SCI patients, there is still limited knowledge about the physiological mechanisms involved during and after sacral nerve stimulation. Important questions remain unanswered, such as how stimuli from the lumbosacral plexus reach the somatosensory cortex, and how the afferent neural pathway is activated under different types of injuries (Spinal Cord Injury Centre of Western Denmark, 2021; Alonso-Calviño et al., 2016). It is also important to know how sequential stimulation over time in the lumbosacral plexus affect the cortical activity (Laufer et al., 2001; Chou and Binder-Macleod, 2007). Finally, it is necessary to investigate any potential side effects resulting from lumbosacral nerve stimulation in the acute and chronical phase of SCIs (Harkema et al., 2011; Lemos et al., 2018; Kasch et al., 2022).

## Cortical response from pelvic nerve stimulation

One of the most intriguing points in the research by Lemos et al. (2023) was the patients’ report of partially recovering tactile sensitivity in different regions of their bodies, previously insensitive. These reports (also described by Possover, 2014) suggest there is a passage of afferent information from the pelvic region to the cortex, especially to the somatosensory cortex.

Even under complete or partial spinal deafferentation, alternative neural pathways, initially dormant, can be reactivated through neuroplasticity, induced by different therapeutic modalities. Recent research on the spinal cord’s dorsal horn has highlighted its critical role in structural reorganization of both local and remote neural networks. This process allows the nervous system to adapt to the loss of sensory input and facilitate functional recovery (Seki et al., 2003; Darian-Smith et al., 2014; Fisher et al., 2018). Moxon et al. (2014), for instance, demonstrated that sensory inputs and neurotrophic factors enable neuroplasticity around the lesion supporting functional healing in sensory pathways even after significant injuries. Additionally, Jones and Pons (1998) and Kaas (2000) highlighted the reorganization of cortical and thalamic sensory maps after injury, underscoring the capacity for adaptive changes in response to altered sensory inputs.

These residual pathways, often underutilized or inactive, may retain a capacity for information conduction that, with appropriate stimulation, can re-establish functional sensory connections. There is evidence that proper electric stimulation below non-responsive regions can result to cortical responses in clinically complete injuries, indicating the presence of residual afferent connections (Awad et al., 2020). By accounting for cortico-cortical modulation, these responses most likely reflect preserved afferent activations through residual somatosensory pathways, referred to as “sensory discomplete” injuries (Dimitrijević, 1988). This type of injury represents an intermediate level between complete and incomplete lesions, with potential for reactivating latent connections.

Pelvic stimulation, as used in the LION procedure, may leverage these pathways by facilitating connections with higher centers, especially the somatosensory cortex (Cardoso Melo et al., 2022). This process would involve both the sprouting of spared afferents and the cortical modulation of sensory inputs, enabling sensory recovery through circuits that would otherwise remain inactive. While this likely results from the activation of residual neural pathways linking these circuits, the lack of sensitivity prior to the LION procedure indicates that the injury initially prevented communication between them (Spinal Cord Injury Centre of Western Denmark, 2021; Alonso-Calviño et al., 2016; Rossignol et al., 2008).

In general, sensory information travels to the spinal cord and is then relayed to sensory centers in the cortex, thalamus, and cerebellum through the dorsal medial lemniscus and the spinocerebellar pathway, respectively. The cerebellum receives somatosensory information and integrates it with a copy of efferent motor commands transmitted by pontine nuclei located in the brainstem to estimate the sensory consequences of movements, where the cerebellum provides feedback to cortical areas through the thalamus (Murray Sherman and Guillery, 2002; Murray Sherman, 2001). On the other side, the motor cortex has loops with different cortical areas, including basal ganglia, cerebellum, and brainstem. The information provided by these loops is used to shape the final motor command, and the output is sent to the neurons of the ventral root of the spinal cord, which command the muscles to generate movement (Shkorbatova et al., 2020; Wenger et al., 2014; Ito, 2006; Courtine et al., 2009). Ozdemir and Perez (2018) and Raineteau and Schwab (2001) observed that, even in injured states, the spinal cord and cortex retain some capacity for adaptive responses, allowing pathways to reorganize and re-establish functional connections when provided with appropriate stimuli.

Therefore, since the intrinsic circuitry of the spinal cord is responsible for the coordination of sensory and motor information, by comparing the changes in power and phase of electrophysiological signals, in people with and without SCI, researchers could gain insights into the neural mechanisms underlying activity in spinal cord injury.

In 2023, Cardoso Melo et al. (2022) proposed a case series protocol to try to stablish a relationship between neuro-electrophysiological activities, via electroencephalography (EEG), and pelvic nerves stimulations (López-Larraz et al., 2015; Pfurtscheller and da Silva, 2017). By analyzing the temporal and spectral patterns of neural activity in patients with SCI, compared to subjects without SCI, specifically focusing on the cortical activity in the sensorimotor area, this preliminary study evaluates the effects of the Possover-LION neuromodulator on these groups (López-Larraz et al., 2015). In this protocol it was evaluated four SCI individuals who had undergone the LION procedure, analyzing their EEG-activity in resting state with the neuromodulator turned on and off, with open and closed eyes.

The study found that the subjects with the LION procedure showed increased activity levels on delta and theta bands and reduced activation levels on alpha and beta bands for both eyes open and closed conditions. These effects were further amplified when the neuromodulator was activated. While further research requires larger sample sizes and correlation assessments with factors like lesion duration, injury level, time with the neuromodulator, and additional physiotherapy treatments, these findings provide initial evidence of EEG rhythm alterations due to a direct LION neuromodulation. The shifts in delta and theta bands, commonly associated with states of neuroplasticity and adaptive changes, indicate the potential for this technique to facilitate cortical reorganization and sensory recovery (Cardoso Melo et al., 2022; Harmony, 2013).

## Discussion

In this review, we outlined the etiology, physiological consequences, and long-term impacts of SCI under neuromodulation therapies. We focused on the potential of the laparoscopic implantation of neuroprosthesis (LION) procedure as an innovative approach for SCI rehabilitation.

Although growing evidence suggests that the LION neuromodulation interventions for SCI patients can improve a range of functions, some challenges relate to the accessibility, affordability, durability, feasibility, and scalability of the approaches still need to be improved. Additionally, the LION procedure often requires individualized programming to account for the specific injury characteristics and functional goals of each patient, which can present challenges in standardizing the approach. Patient variability, including differences in injury level, completeness, and chronicity, also influences response to stimulation, necessitating personalized adjustments to optimize outcomes. More critically, a more in-depth understanding of mechanisms is also crucial.

The main difference between the LION-based strategy and the other strategies available and tested is its unique combination of FES-like directly induced movements and chronic stimulation, which can potentially produce neuroplasticity effects similar to that observed in spinal cord stimulation protocols.

Stimulation of lumbosacral nerves, specifically the femoral, sciatic and pudendal nerves, can activate different sensory and motor cortical regions. However, different types of spinal cord injuries can produce different patterns of rhythmic activity in the cortex and different types of desynchronizations. Each pattern of activity can impact how the brain adapts to the type of injury and rehabilitation therapies. Neuromodulation has been shown to result in gains in muscle mass, improved control of urinary and bowel sphincters, and increased mobility using pelvic gait.

Lemos et al. (2023) demonstrated how the LION procedure, conjugated with rehabilitation, can rescue different physiological functions of patients with spinal cord injury. The procedure had a significant impact on mobility measures, with all patients showing improvement in mobility and most patients able to initiate gait training.

Observations also revealed that patients with complete SCI experienced measurable recovery in tactile sensitivity after undergoing the LION procedure. Additionally, patients with incomplete spinal cord injury have been found to experience a reclassification of the ASIA sensory scores. These results suggest there must be some flow of ascending information from the stimulated nerves to the somatosensory cortex. Overall, these findings highlight the potential benefits of neuromodulation in improving sensory and motor function in patients with SCI.

Another potential extension of the LION procedure is its integration with brain-computer interface (BCI) approaches (Maiseli et al., 2023; Lebedev and Nicolelis, 2006; Yin et al., 2023). This integration could eliminate the need for the remote-control commands (of the neuromodulators) that is currently used to switch between programs, creating a direct link between the decoding of neural signals related to motor intention and sensory feedback (Wenger et al., 2014; Bockbrader, 2019). Real-time monitoring of neural signals can potentially open the way for new closed-loop feedback mechanisms, where stimulation parameters are dynamically adjusted based on the individual’s neural activity and motor intentions. This closed-loop dynamics may optimize rehabilitation outcomes by tailoring interventions to the specific needs and progress of each patient, offering more natural and intuitive control over movements (Alam et al., 2016; Sun and Morrell, 2014). Finally, the improved tactile sensitivity reported after the LION procedure could be further leveraged by incorporating BCI-generated sensory feedback (Lundell et al., 2011; Fong et al., 2021). In this way, the combined approach may create a synergistic environment for enhanced neuroplastic changes, potentially accelerating the recovery process.

The LION procedure represents a promising avenue for advancing SCI rehabilitation. This approach has the potential to revolutionize treatment strategies, offering more personalized, adaptive, and effective rehabilitation for individuals with SCI. Addressing these limitations through advanced programming algorithms and real-time adaptive feedback systems may be promising for broader applicability. Continued research and clinical trials are crucial to determine the feasibility and enhance the synergistic effects of this innovative technique.

## Footnotes

^1^Although there are other electrical stimulation techniques for lumbosacral nerves, in this article we will focus on describing the results using the LION technique specifically.
